# Leveraging survival analysis and machine learning for accurate prediction of breast cancer recurrence and metastasis

**DOI:** 10.1038/s41598-025-87622-3

**Published:** 2025-01-29

**Authors:** Shahd M. Noman, Youssef M. Fadel, Mayar T. Henedak, Nada A. Attia, Malak Essam, Sarah Elmaasarawii, Fayrouz A. Fouad, Esraa G. Eltasawi, Walid Al-Atabany

**Affiliations:** 1 Center for Informatics Science (CIS), School of Information Technology and Computer Science, Nile University, 26th of July Corridor, Sheikh Zayed City, Giza, 12588 Egypt; 2 Baheya Center for Early Detection and Treatment of Breast Cancer, Research Center, Giza, 12511 Egypt

**Keywords:** Breast cancer, Recurrence prediction, Machine learning, Metastasis, Survival analysis, Cancer, Computational models, Risk factors, Cancer, Breast cancer

## Abstract

Breast cancer, with its high incidence and mortality globally, necessitates early prediction of local and distant recurrence to improve treatment outcomes. This study develops and validates predictive models for breast cancer recurrence and metastasis using Recurrence-Free Survival Analysis and machine learning techniques. We merged datasets from the Molecular Taxonomy of Breast Cancer International Consortium, Memorial Sloan Kettering Cancer Center, Duke University, and the SEER program, creating a comprehensive dataset of 272, 252 rows and 23 columns. Our methodology utilized three predictive strategies: assessing recurrence risk, differentiating local from distant recurrences, and identifying potential metastatic sites. Key prognostic factors were identified through survival analysis. LightGBM, XGBoost, and Random Forest models were employed and validated against data from the Baheya Foundation. The models demonstrated strong performance; the survival analysis achieved a C-index of 0.837. The LightGBM model reached an AUC of 92% in predicting recurrences, while XGBoost and Random Forest models distinguished recurrence types with up to 86% accuracy, and they effectively differentiated between bone metastasis and all other locations combined (brain, liver, and lungs). This study highlights the significant potential of machine learning in advancing breast cancer management and sets a new benchmark for predictive analytics. Future research will integrate genetic data to further enhance these models.

## Introduction

Breast cancer is a significant health issue worldwide, characterized by the uncontrolled growth of abnormal cells in breast tissue that form tumors^1^. In 2020, approximately 2.3 million women were diagnosed with breast cancer, and 685,000 deaths were reported globally^2^. By the end of the same year, an estimated 7.8 million women worldwide were living with a breast cancer diagnosis received within the past five years, making it the world’s most prevalent cancer^2^. Breast cancer is the most common cause of cancer mortality of women in Egypt, with over 22,000 new cases diagnosed annually, accounting for 33% of female cancer cases^3^. A comparison between the Gharbiah Cancer Registry (GCR) and the U.S. SEER Program database revealed that GCR cases are a decade younger, with 19% aged above 40, and are diagnosed at more advanced stages compared to SEER cases, which are diagnosed at stage I, II, III, or IV^4^.This significant difference in the stage at diagnosis indicates disparities in early detection and access to healthcare, which are critical areas for improvement. Baheya Foundation stands out as a beacon of hope and support for breast cancer patients in Egypt. Baheya^5^ had achievements such as serving over 247,942 patients, administering 121,219 chemotherapy sessions, conducting 279,000 radiotherapy sessions, and performing 20,384 surgeries, Baheya Foundation continues to be a vital resource in the battle against breast cancer in Egypt.

Recurrence in breast cancer refers to the reappearance of cancer cells in the breast or nearby tissues after a period of apparent remission, which can occur months or even years after the completion of treatment. Global research indicates that around 30% of women experience a recurrence after the primary treatment for breast cancer^6^. There are two types of recurrence based on where it recurs: local and distant. Local recurrence means the cancer returns to the same breast or chest area as the original tumor. Distant recurrence, also known as metastatic or Stage 4 breast cancer, occurs when the cancer spreads away from the original tumor to the lungs, bones, brain, liver, or other parts of the body^7^.

The most recent researches in breast cancer prognosis are presented here, with a focus on enhanced survival analysis methods and the use of deep learning and machine learning to predict metastasis and recurrence. The variability in efficacy resulting from feature selection and model architecture choices is also highlighted. Jung et al.^8^ developed algorithms from real-world health data, achieving 94.2% sensitivity and 79.2% positive predictive value. Their Kaplan-Meier and Cox regression models for RFS closely matched chart-reviewed data, emphasizing key factors such as Human Epidermal Growth Factor Receptor 2 (HER2) status, tumor grade, and stage. Clift et al.^9^ compared regression-based models and machine learning for breast cancer prognostication. Cox proportional hazards models outperformed machine learning, with a Harrell’s C index of 0.858. This underscores the effectiveness of regression methods for clinical risk stratification and prognosis in breast cancer patients. Survival analysis has also been utilized for feature selection across multiple data modalities, extending beyond clinical and tabular features to include radiomics and imaging data. Wu et al.^10^ proposed a multi-task learning approach combining survival prediction with semi-supervised tumor segmentation using a shared Transformer encoder. Tested on two local hospital datasets, it achieved comparable segmentation results and acceptable survival prediction accuracy, highlighting the potential of integrating feature extraction into survival analysis. Zhang et al.^11^ utilized survival analysis to develop a diffusion-weighted imaging-based radiomics signature for predicting progression-free survival (PFS) in muscle-invasive bladder cancer patients. The radiomics nomogram demonstrated superior survival prediction performance (C-index: 0.702) compared to traditional models.

Recent studies have investigated a wide range of machine learning methods, including logistic regression, RF, support vector machine (SVM) classification, XGBoost, and decision trees, in order to predict the recurrence of breast cancer. AdaBoost offers the best prediction performance, according to Zuo and Yang^12^, with a recall rate of 94%. In order to estimate the risk of recurrence for patients with breast cancer after surgery, Zeng et al.^13^ extracted clinicopathological features from unstructured clinical electronic health records. With the help of LSTM, XGBoost, and SVM, they got good outcomes. With 89% recall, The LSTM model exhibited superior performance compared to other models, demonstrating its effectiveness in predicting breast cancer recurrence and metastasis.

Chakkouch^14^ aimed to predict the types of breast cancer recurrence (Local, distant, regional) by comparing the performance of various machine learning techniques, including logistic regression, decision tree, K-Nearest Neighbors (KNN), and Neural networks (NN). They utilized a dataset comprising clinical and pathological data from 1189 patients treated between 2015 and 2022 in Meknes, Morocco. The ANN model outperformed the others, achieving the highest accuracy of 91%. For the metastasis location, Audrey Shiner^15^ aimed to predict patterns of distant metastasis (DM) in breast cancer patients. Results showed that most patients (57%) developed bone metastases. Additionally, the study suggests that clinicopathologic and treatment variables used in machine learning prediction models can effectively predict the first site of metastasis in breast cancer. Zhong^16^ utilized a retrospective analysis of breast cancer patients from the SEER database to construct diagnostic and prognostic models employing six machine learning classifiers. The XGBoost algorithm outperformed others, achieving an AUC of 98% for diagnosis and 88% for prognosis. Recent research has identified critical risk factors and influential features associated with bone metastasis in breast cancer. These studies provide insights into hormone receptor statuses and breast cancer subtypes as significant predictors of bone metastasis^17–20^. Further advancements highlight the potential of integrating radiomics and deep learning to predict distant metastasis by leveraging multimodal data, including imaging features and molecular insights^21–24^.

Despite significant progress in using machine learning for breast cancer prognosis, several gaps remain in the current research. The state of the art studies utilize limited datasets, which restricts the generalizability of their models. There is often a lack of comprehensive external validation, particularly across diverse geographic and demographic populations. Additionally, feature selection processes may not fully account for the complex interactions among clinical factors, potentially limiting the accuracy of predictions. Addressing these gaps requires the use of more robust and diverse datasets, thorough validation across different populations, and refined feature selection methods to improve the reliability and applicability of predictive models in clinical settings.

In this study, survival analysis and machine learning are employed to develop advanced predictive models that provide insights into the timing and key prognostic factors of breast cancer recurrence. The methodology focuses on three main predictive strategies: first, the risk of recurrence is evaluated through survival analysis; second, local recurrences are distinguished from distant metastases; and third, the specific locations of metastatic recurrence are pinpointed. By extensively utilizing clinical data, the accuracy of predictions is ensured. A crucial aspect of the research is the rigorous validation of these models using real-world data from Egyptian breast cancer patients, sourced from the Baheya Foundation, to confirm their effectiveness and applicability within the local healthcare setting.

## Results

### Recurrence-free survival analysis

**Fig. 1:**
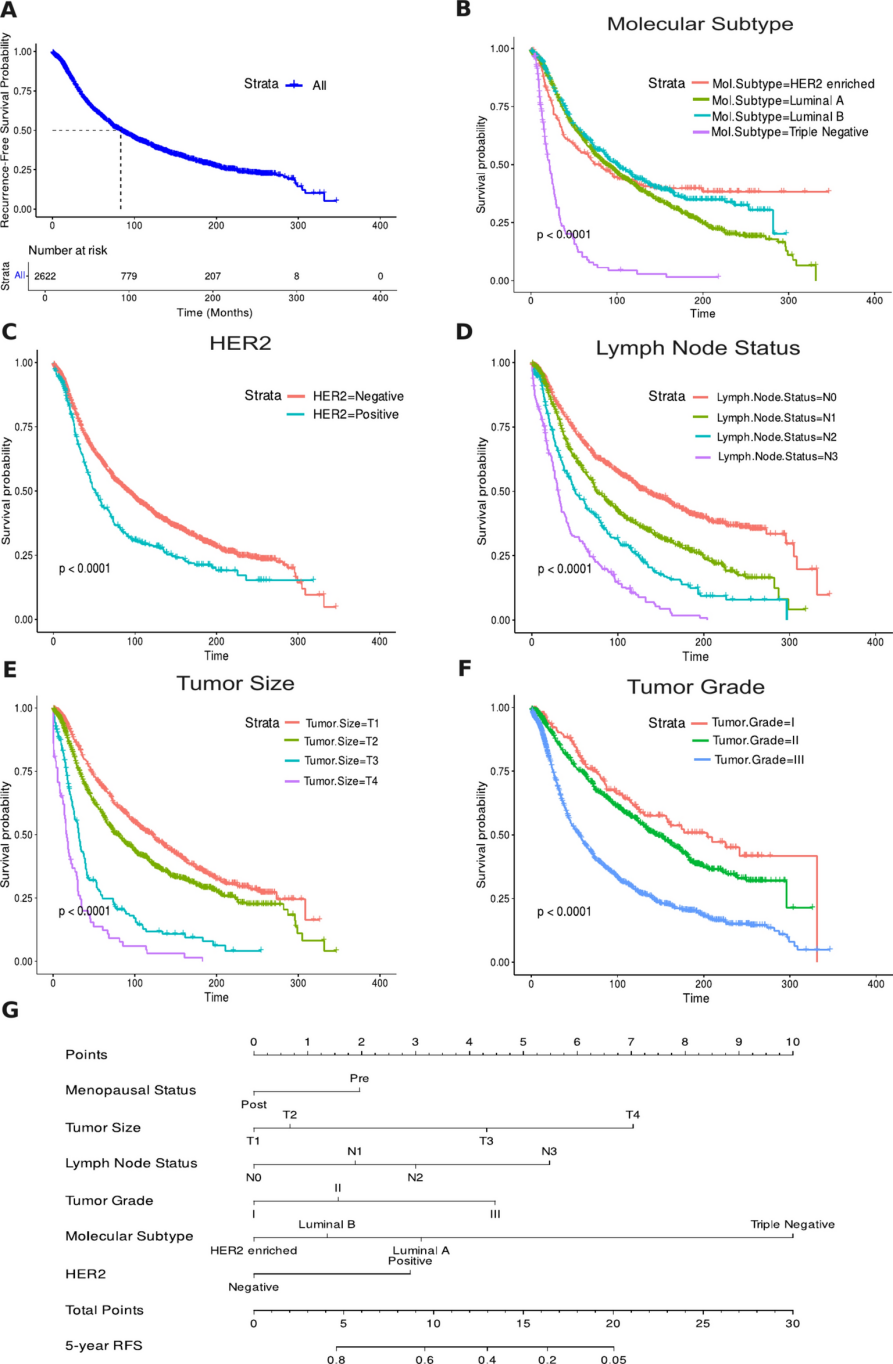
Recurrence-free survival analysis. (**A**) Kaplan-Meier RFS curve. Kaplan-Meier survival plots adjusted for study covariates: (**B**) Molecular Subtype, (**C**) HER2, (**D**) Lymph Node Status, (**E**) Tumor Size, (**F**) Tumor Grade. (**G**) Nomogram for predicting RFS.

The Kaplan-Meier curve displayed a RFS rate of 59.2% (Fig. 1A). Kaplan-Meier analyses were conducted for each covariate to visualize survival curves (Fig. 1B–F). Log-rank tests and univariate Cox regression analyses revealed significant differences in survival rates across various factors (all *p*<0.001), with menopausal status, tumor size, lymph node involvement, tumor grade, molecular subtypes, histological type, hormone receptor status, and overall survival status (month) influencing RFS. Tumor location had no significant effect on recurrence risk (*p* = 0.451).

In the multivariate Cox regression analysis in Table 1, several factors significantly influenced recurrence risk. Premenopausal women had a higher risk than postmenopausal women. Tumor grade was significant, with Grade II and Grade III tumors showing higher risk than Grade I. Triple Negative breast cancer posed the highest risk among molecular subtypes. Larger tumors and lymph node involvement also increased risk. Hormone receptor status was crucial; ER-positive status lowered risk, while HER2-positive status raised it. Overall survival status was significantly associated with recurrence risk. Conversely, tumor location, histological type, and Progesterone receptor (PR) status did not show significant associations with recurrence risk in this cohort. The predictive accuracy of the Cox regression model was assessed using a C-index of 0.837. A nomogram was developed based on the Cox regression model, providing a practical tool for clinicians to estimate individual recurrence risk and tailor treatment strategies accordingly (Fig. 1G).

**Table 1:** Univariate and multivariate cox regression analysis of RFS.

Covariate	Univariate		Multivariate	
	HR (95% CI)	*P*-value	HR (95% CI)	*P*-value
Menopausal status				
Post	Reference		Reference	
Pre	1.798 (1.618, 1.998)	<0.001	1.697 (1.514, 1.903)	<0.001
Tumor size				
T1	Reference		Reference	
T2	1.321 (1.178, 1.481)	<0.001	1.024 (0.908, 1.155)	0.700
T3	3.147 (2.604, 3.802)	<0.001	1.599 (1.304, 1.961)	<0.001
T4	6.100 (4.726, 7.874)	<0.001	2.573 (1.968, 3.364)	<0.001
Lymph node status				
N0	Reference		Reference	
N1	1.535 (1.355, 1.739)	<0.001	1.268 (1.115, 1.443)	<0.001
N2	2.274 (1.944, 2.660)	<0.001	1.441 (1.218, 1.706)	<0.001
N3	3.887 (3.270, 4.620)	<0.001	2.075 (1.724, 2.496)	<0.001
Tumor grade				
I	Reference		Reference	
II	1.288 (0.9999, 1.660)	0.050	1.382 (1.070, 1.785)	0.013
III	2.654 (2.0843, 3.378)	<0.001	2.039 (1.590, 2.615)	<0.001
Mol. subtype				
HER2 enriched	Reference		Reference	
Luminal A	1.118 (0.9254, 1.350)	0.248	2.262 (1.627, 3.146)	<0.001
Luminal B	0.9454 (0.7651, 1.168)	0.603	1.789 (1.292, 2.478)	<0.001
Triple Negative	4.564 (3.4850, 5.978)	<0.001	2.861 (2.058, 3.978)	<0.001
Histological type				
Infiltrating duct and lobular carcinoma	Reference		Reference	
Infiltrating duct carcinoma	1.467 (1.2109, 1.777)	<0.001	1.099 (0.902, 1.338)	0.348
Invasive Breast Carcinoma	1.045 (0.3856, 2.833)	0.931	0.508 (0.186, 1.386)	0.186
Lobular carcinoma	1.736 (1.3560, 2.223)	<0.001	1.270 (0.988, 1.632)	0.062
ER				
Negative	Reference		Reference	
Positive	0.5441 (0.4683, 0.6321)	<0.001	0.703 (0.508, 0.974)	0.034
PR				
Negative	Reference		Reference	
Positive	0.8051 (0.7209, 0.8991)	<0.001	0.998 (0.870, 1.144)	0.976
HER2				
Negative	Reference		Reference	
Positive	1.445 (1.247, 1.673)	<0.001	1.489 (1.219, 1.819)	<0.001
Tumor location				
Left	Reference		Reference	
Right	1.041 (0.9375, 1.156)	0.451	0.995 (0.895, 1.106)	0.924
Overall survival status (Month)	0.972 (0.9702, 0.9737)	<0.001	0.972 (0.970, 0.974)	<0.001

### Prediction insights of recurrence and baheya’s validation

**Fig. 2:**
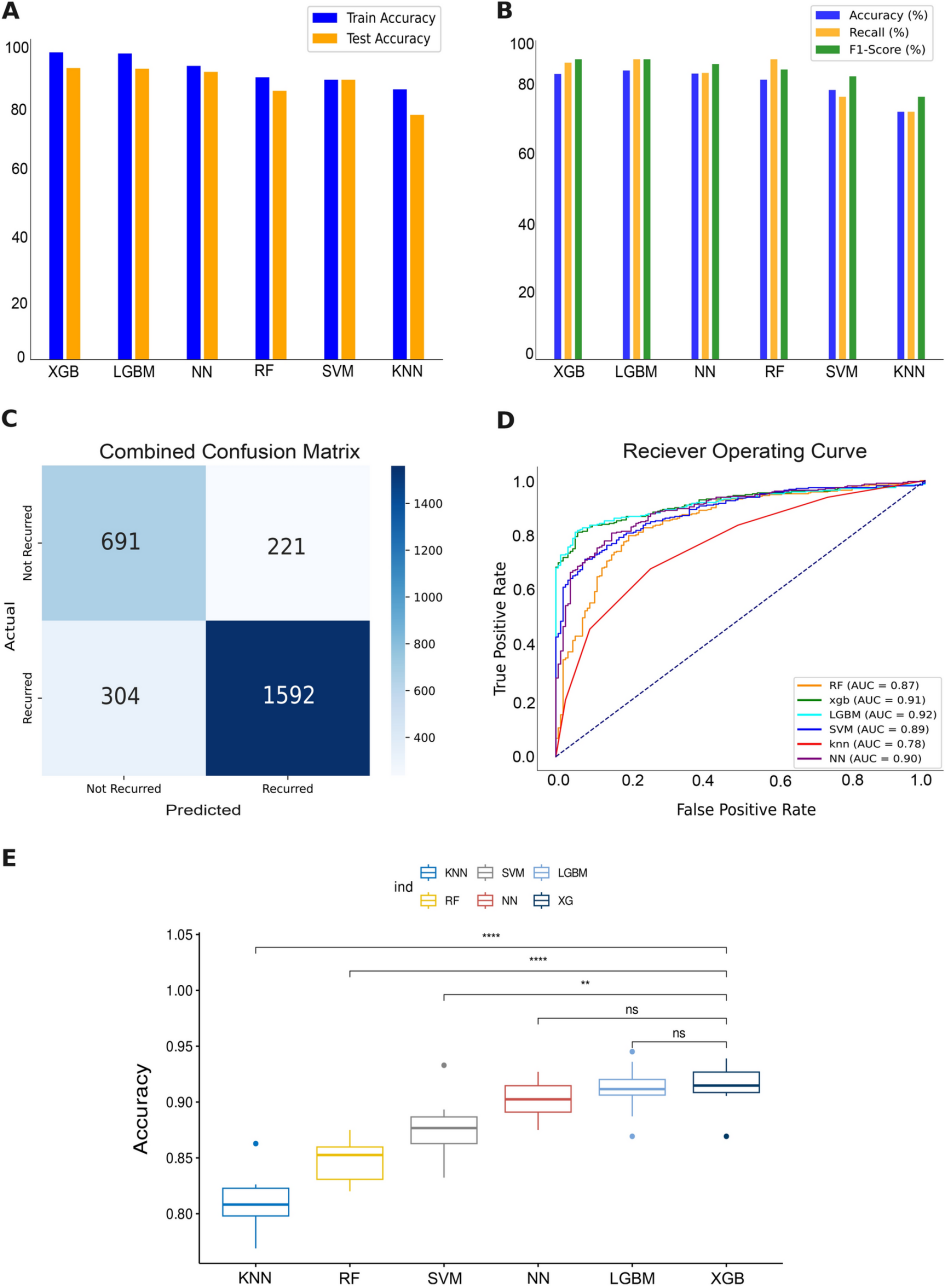
Recurrence vs. not recurrence approach results on validation set. (**A**) shows differences in training and testing accuracies of 6 machine learning models. (**B**) reveals the performance of the evaluation metrics: Accuracy, Recall, and F1 score. (**C**) combines the confusion matrices of all 6 models. The confusion matrix displays the predicted classes on the X-axis and the true classes on the Y-axis, with the color of the diagonal blocks illustrating the closeness of the match between the predicted and the true class. The darker the blue color of the diagonal line, the better the model prediction accuracy. (**D**) is a combined ROC curve for all 6 models. (**E**) visualizes statistical p-value results with cross-validation of each model.

For the Recurrence approach, the **training and testing** results in (Fig. 2A) of various machine learning models reveal their efficacy in predicting breast cancer recurrence. During training, XGBoost achieved a mean accuracy of 91.5%, followed closely by LGBM and NN with mean accuracy of 91.3%, and 90% respectively. SVM and RF models demonstrated lower performances, achieving mean accuracy of 87% and 84.7% respectively. Finally the least performing model was the KNN having a mean accuracy of 81%. In testing, all models maintained high accuracies, ranging from 79.45% to 92%, underscoring their generalization capabilities.

The results from the models **validation** achieved accuracy range from 74 to 84% as shown in Table 2. These results were generated with the validation set of real patient data of Egyptian population obtained from Baheya Foundation. Apart from the accuracy metric, other evaluation metrics were also calculated such as recall (Fig. 2B), f1 score (Fig. 2B), and ROC-AUC (Fig. 2D). Recall was utilized due to its crucial role in identifying positive cases, which directly influences patient survival rates. (Figure 2C) highlights the misclassification biases that were assessed in the confusion matrix combined for all our models based on the validation dataset. However, there were misclassifications where (25.87%) of the patients who were actually not experiencing a recurrence were incorrectly classified as experiencing a recurrence, and (17.61%) of the patients who were actually experiencing a recurrence were incorrectly classified as not experiencing a recurrence. As well as according to the ROC-AUC combined as shown in (Fig. 2D), XGBoost and LGBM have the highest AUC of 92% indicating their effectiveness in identifying instances correctly. As shown in (Fig. 2E) the results of wilcoxon test present the significant differences between KNN and XGBoost in accuracy. moreover, the LGBM and XGBoost have no significant differences in the accuracy.

### Prediction of recurrence types and validation with baheya’s dataset

**Fig. 3:**
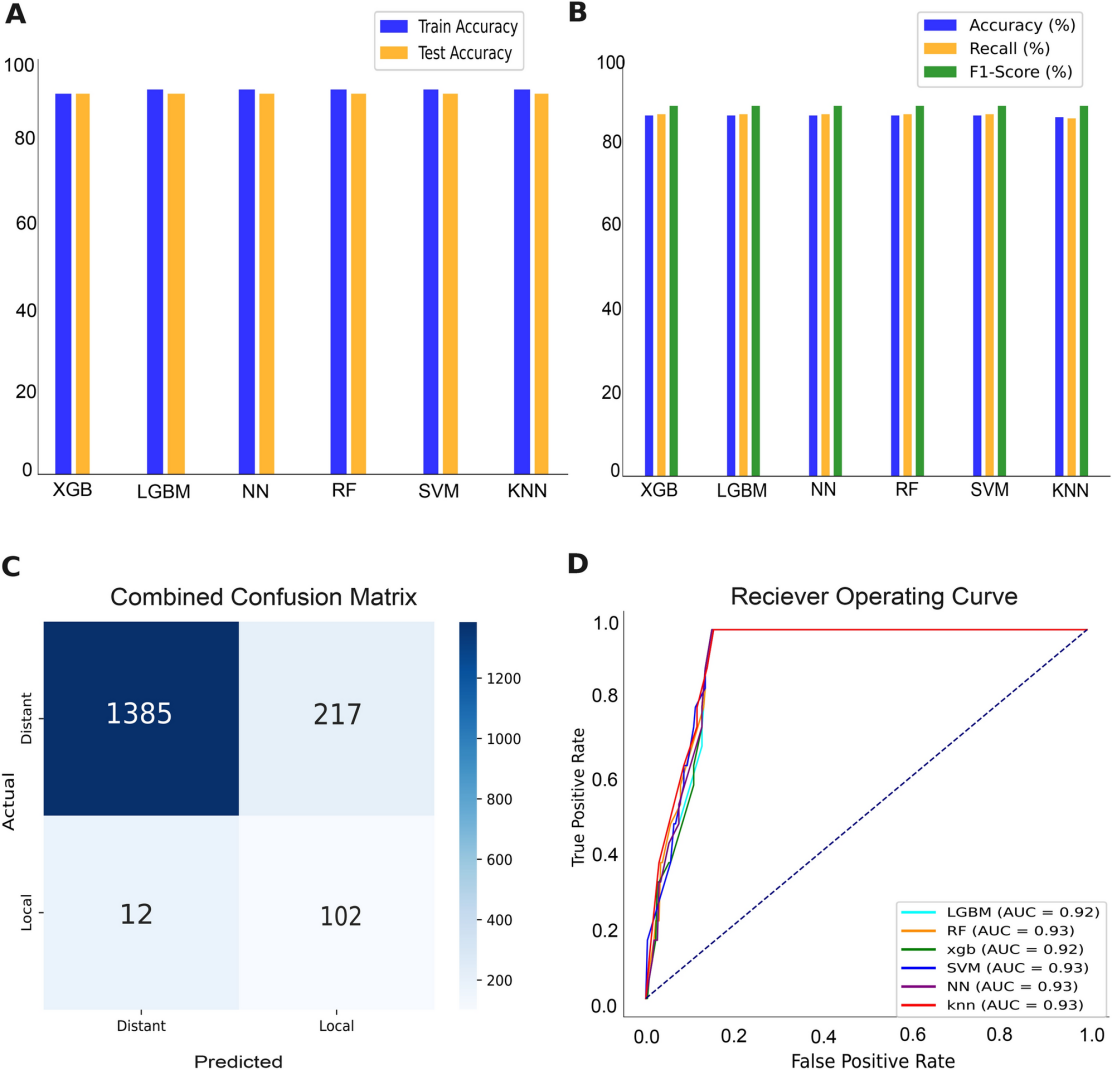
Local vs. distant recurrence results on the validation set. (**A**) shows slight differences in training and testing accuracies of 6 machine learning models. (**B**) reveals the performance of the evaluation metrics: Accuracy, Recall, and F1 score. (**C**) combines the confusion matrices of all 6 models. The confusion matrix displays the predicted classes on the X-axis and the true classes on the Y-axis, with the color of the diagonal blocks illustrating the closeness of the match between the predicted and true class. The darker the blue color of the diagonal line, the better the model prediction accuracy. (**D**) is a combined ROC curve for all 6 models.

The **training and testing** results of six machine learning models demonstrate their efficacy in predicting the type of recurrence. As presented in (Fig. 3A), the models achieved approximately similar results during both the training and testing phases, with an accuracy around 90% in both cases, except for the KNN model, which achieved an accuracy of 89% during testing. Additionally, during testing, the recall and F1-score for all models were near to 90%. Overall, all models performed comparably on the data, and the consistently high accuracy, recall, and F1 scores demonstrate the models’ effectiveness yielding robust results.

The **validation** results of All models demonstrated a performance accuracy around 86%, The evaluation metrics as shown in Table 2 indicate that the models performed similarly across most metrics. Notably, KNN model exhibited a slightly lower by one percentage point compared to the other models. Apart from the accuracy metric, as shown in (Fig. 3B), the percentage of recall, F1-score are very close between models of values 87% and 89% respectively. The combined confusion matrix for all models (Fig. 3C) revealed a misclassification percentage of approximately 13.55% for the Distant class and 10.53% for the Local class. As well as according to the ROC-AUC values of all models were closely aligned, with scores of 92% and 93% indicating a consistently high level of performance across the models as shown in (Fig. 3D). Finally, the external validation results further corroborated the effectiveness of these models, demonstrating very good performance on real patient data.

### Prediction of distant recurrence sites

**Fig. 4:**
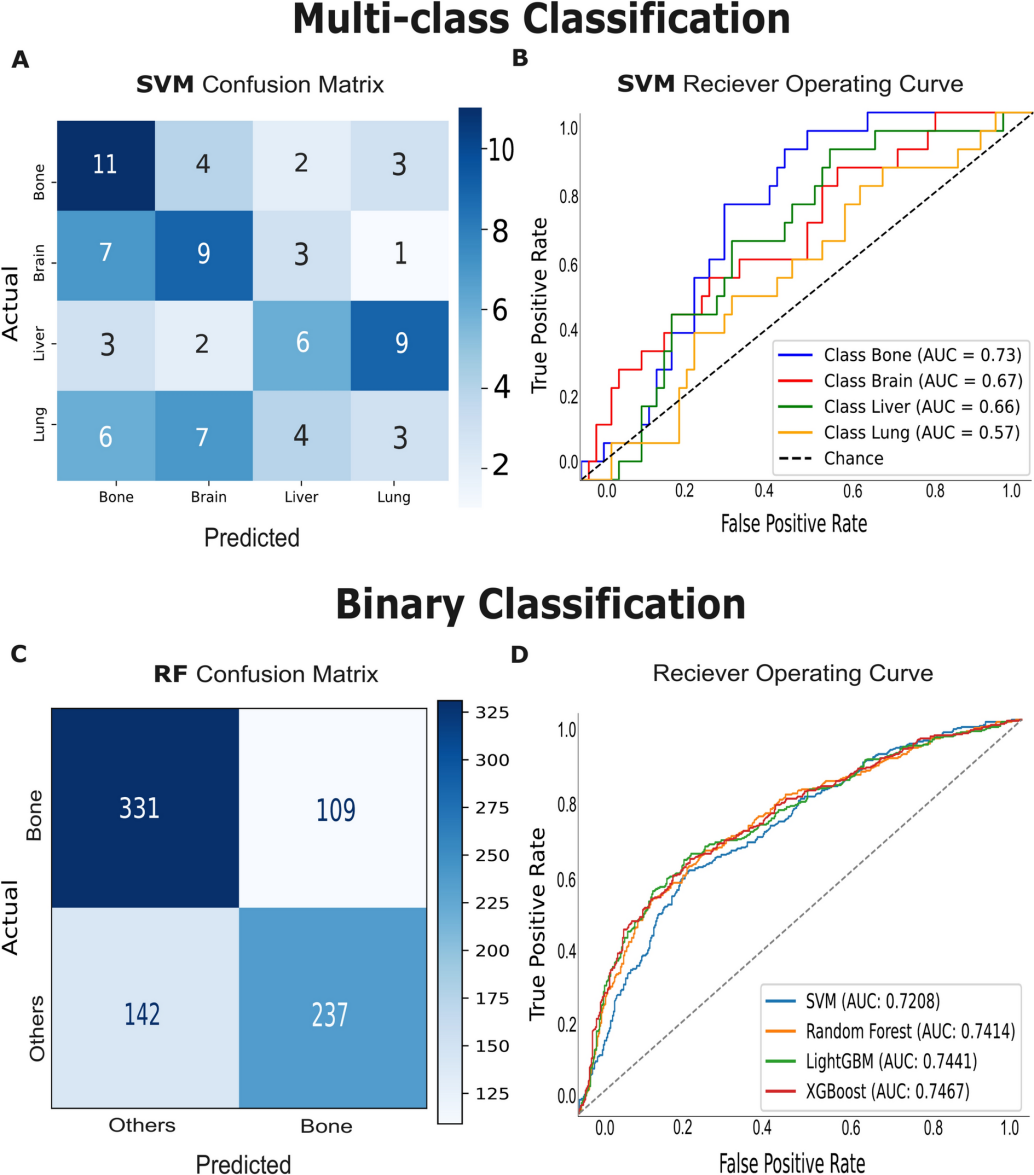
Distant sites prediction results on test set. (**A**) Multi-class classification SVM confusion matrix showing accuracy distribution across multiple classes. (**B**) ROC curve of the SVM model, displaying the AUC values for bone, lung, liver, and brain metastasis predictions. (**C**) Confusion matrix for binary classification distinguishing between ’Bone’ and ’Other’ locations. (**D**) ROC for the binary classification model, highlighting its performance in differentiating between the two categories.

For the multiclass classification task, the SVM model demonstrated high performance in predicting bone metastasis but significantly lower performance for the other classes (brain, liver, and lung). The F1-scores for each class were as follows: bone 47%, brain 43%, liver 34%, and lung 17%, indicating a strong prediction of bone location. As shown in the confusion matrix (Fig. 4A), the bone class exhibited the lowest misclassification rate, highlighting the model’s ability to distinguish bone metastases from other sites. However, significant misclassifications were observed for the brain, liver, and particularly lung, which had the highest rate of incorrect predictions. The model struggled to differentiate between lung, liver, and brain classes. The ROC curve (Fig. 4B) further supports this observation, with the highest AUC (0.73) achieved for bone metastasis.

For the results of the models distinguishing bone metastases from all other metastatic locations combined. As shown in Table 2, the models achieved close testing accuracies, with an average value of 74%, except for the SVM model, which had the lowest accuracy at 69%. The F1-scores across the models were similar, with LightGBM achieving the highest value of 70%, while the other models remained around 69%. The confusion matrix for the Random Forest model in (Fig. 4C) demonstrates that the model correctly classified most of the bone cases, but exhibited higher misclassification rates for the Others class, reflecting a challenge in correctly predicting non-bone metastases. In the ROC as shown in (Fig. 4D), XGBoost, Random Forest, and LightGBM achieved high and closely aligned AUC values of 0.74. However, the SVM model had the lowest AUC of 0.72, consistent with its relatively lower performance in accuracy and F1-score.

#### Data distribution insights

**Fig. 5:**
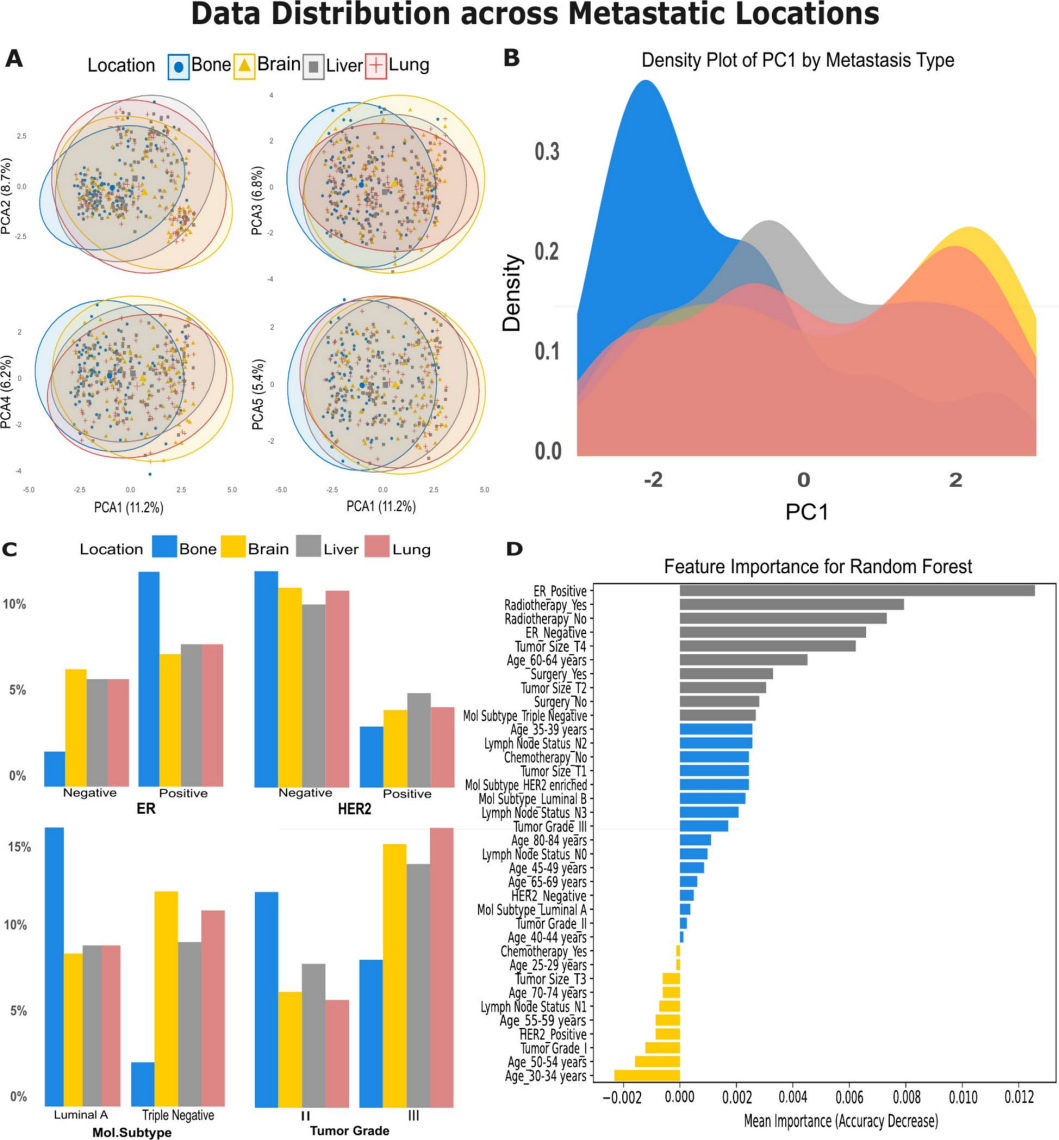
Features distribution across metastatic locations. (**A**) PCA distribution of metastatic cancer data showcasing the variance explained by the first principal component (PC1) across different metastasis types (Bone, Brain, Liver, lung). (**B**) Denisty plot that illustrates the overlapped clusters between different locations. (**C**) Histograms showing the distribution of tumor characteristics and treatment modalities across different subtypes and responses, such as hormone receptor status (ER, HER2 ±), tumor size (T1, T2, T3, T4), molecular subtype (Luminal A, Luminal B), and treatments received (Chemotherapy, Radiotherapy). (**D**) SHAP feature importance.

In the PCA plots (Fig. 5A), the first principal component (PC1) captures 11.2% of the variance, while subsequent components (PC2, PC3, and PC4) account for smaller proportions of variance. As observed, the clusters of lung, liver, and brain locations overlap, suggesting that they will be difficult to separate, while the bone metastases exhibit slightly more distinct clustering compared to the other metastatic sites, indicating some degree of differentiation. In the density plot of PC1 (Fig. 5B), bone metastases show a distinct peak in the distribution, while the density plots for brain, liver, and lung metastases overlap in an obvious way.

In the feature categories distribution (Fig. 5C), each bar chart represents the distribution of features across different metastatic locations, providing insights into potential differences between them. As observed, features such as ER status, molecular subtype, tumor grade, and HER2 status show very similar distributions among lung, liver, and brain metastases, indicating small variation between these locations. In contrast, bone metastasis shows a distinct distribution pattern for these features, highlighting notable differences compared to the other locations. These features can be observed in the SHAP feature importance for a Random Forest model (Fig. 5D), it shows that Features like “ER Positive”, “Mol Subtype Triple negative”, “ER Negative” and “Tumoe Size T4” are highly influential in predicting the target variable.

**Table 2:** Performance of machine learning classifiers in predicting recurrence, types of recurrence, and distant metastasis locations for validation cohort.

Classifier	Accuracy (%)	Recall (%)	F1-Score (%)
Recurrence			
LGBM	84	89	89
XGBoost	82	86	87
NN	83	83	87
SVM	80	78	84
RF	83	94	89
KNN	74	75	80
Types of recurrence			
LGBM	86.75	87	89
XGBoost	86.70	87	89
NN	86.77	87	89
SVM	86.71	87	89
RF	86.72	87	89
KNN	86.36	86	89
Distant metastasis locations (Bone Vs. Others)			
LGBM	74	70	70
XGBoost	74	70	69
SVM	69	69	69
RF	74	69	69

### Comparative analysis with previous studies

Supplementary Table S2 presents a comparative analysis of the findings against previous studies. While the performance metrics of the models show some variation compared to earlier research, these differences can be explained by several significant factors. This study utilized a notably larger dataset, with 4,690 and 22,205 samples for recurrence binary classification and recurrence types prediction, respectively, compared to 342 and 1,189 samples in the prior studies by Zuo and Yang^12^ and Chakkouch^14^. Additionally, rigorous external validation sets were employed, enhancing the robustness and generalizability of the results. For instance, the model for distant recurrence sites prediction achieved an AUC of 0.86 with 13,400 samples, compared to Zhong’s^16^ AUC of 0.80 with a sample size of 3492. These improvements enhance evaluation, demonstrating the approach’s effectiveness in recurrence prediction.

## Discussion

The early detection of breast cancer recurrence, whether local or distant, is crucial for optimizing patient management and improving outcomes. Early prediction enables tailored treatment strategies that can significantly enhance survival rates and patient well-being. In this regard, Artificial Intelligence (AI) plays a vital role in healthcare by improving the accuracy and efficiency of predictive models. Our study leverages AI to refine the prediction of breast cancer recurrence, allowing for timely and personalized interventions. AI’s ability to process large datasets and extract meaningful patterns offers substantial advantages over traditional methods, to not only improves diagnostic accuracy but also sets the stage for a new era of patient-centered medical care. The survival analysis revealed key insights into the factors influencing breast cancer relapse. Initial findings from the univariate Cox regression model identified “Tumor Location” as an insignificant predictor of recurrence. This finding aligns with Jung et al.^8^, who also identified tumor location as a less critical factor when considered in isolation. In the multivariate Cox regression analysis, PR and Histological Type also emerged as insignificant predictors. When comparing these results to those reported by Jung et al., who developed algorithms for predicting breast cancer recurrence using real-world health data, common risk factors arise. Jung et al. found tumor stage, tumor grade, HER2 status, menopausal status, tumor size, and lymph node involvement to be significant predictors of recurrence. Similarly, this study found that tumor grade, HER2, menopausal status, tumor size, lymph node involvement, and overall survival status were significant predictors in the multivariate model. This alignment in findings reinforces the importance of these risk factors across different cohorts. However, the discrepancies in other factors could be attributed to differences in the study design or the underlying data used. The non-significance of “PR” and “Histological Type” in the multivariate model underscores the importance of considering the interactions among clinical features. These findings suggest that while some factors may appear influential in isolation, their predictive power can diminish in a more comprehensive model. During the feature selection process, features commonly identified in state-of-the-art studies were initially included, as referenced in papers^12,15^, and^16^. This initial selection encompassed well-established predictors of breast cancer recurrence such as tumor grade, HER2, ER, PR, menopausal status, tumor size, lymph node involvement, Histological Type, and Tumor Location. While this approach was grounded in existing literature, the resulting model performance was only intermediate. To enhance the predictive accuracy of the models, the approach was refined by selecting features that demonstrated significant associations in the survival analysis of breast cancer relapse events. Focusing on the most impactful predictors led to a marked improvement in the model’s performance. By relying exclusively on features with high predictive power, the models became more reliable and accurate in forecasting breast cancer recurrence. The exploration began with the Recurrence Approach, demonstrating the substantial application of advanced machine learning methodologies. Results showcased a high AUC of 92% for LGBM on the external validation dataset. In contrast, Zuo and Yang^12^ explored eleven machine learning algorithms with a different set of features, such as CEA, CA125, fibrinogen, and tumor diameter. Their study highlighted the AdaBoost algorithm, which achieved a superior AUC of 98.7%. Building on this success, the study delved into the Recurrence Types Prediction Approach. Comparing the results with those of Chakkouch^14^, several key differences and advantages emerged. Models achieved accuracies ranging from 89% to 90% during testing. In contrast, the referenced study reported accuracies ranging from 84.6% to 91%. Both studies employed various machine learning models, including KNN and NN. However, the use of a significantly larger and more generalized dataset of 22,205 patients, compared to their 1,189 patients, provided a more comprehensive and robust analysis. Regarding the prediction of the metastatic cancer locations among four distinct sites: bone, brain, liver, and lung, a multi-class classification approach was utilized. However, the predictive accuracy of this model was less than optimal, predominantly because metastasis to the bone is more prevalent and typically occurs earlier than in other locations, as detailed by Ye^17^. This prevalence led to a disproportionately high predictive probability for bone metastasis. To gain deeper insights, principal component analysis (PCA) and density plots were employed to examine the data distribution and interaction among the various metastatic sites. These analyses revealed distinct separability of the bone from other locations, while the other sites (brain, liver, and lung) showed significant overlap, making them impossible to tell apart within the parameters of the model. Further analysis based on the feature importance and distribution across samples revealed significant factors influencing bone metastasis. Both ER positive and ER negative statuses have been identified as impactful, with Wei et al.^19^ highlighting their importance in bone metastasis. Remarkably, tumor size T4 emerged as the most influential, aligning with findings from Lui^18^ which suggest that increased tumor size correlates with a heightened risk of bone metastasis. Additionally, Wei’s^19^ research indicates that the luminal A subtype is predominantly associated with bone metastasis, whereas Otto identifies the luminal B subtype as having the highest incidence risk for bone metastasis^20^, a finding that is corroborated by our study. Other significant features affecting bone metastasis include radiotherapy and tumor grade III. These features are consistent with the risk factors identified in Liu’s study^18^, which supports their relevance in predicting bone metastasis. Furthermore, all these features align with the survival analysis performed in our study to identify risks of recurrence-free survival, indicating similar pathological patterns observed in both metastasis and recurrence, confirming the consistent influence of these risk factors across different manifestations of the disease. Due to these findings, the strategy was shifted to develop a binary classification model that differentiates between bone metastasis and all other locations combined. This adjustment significantly enhanced the model’s performance, reflecting the distinct pathological and metastatic characteristics of bone relative to other metastasis sites. This model differentiates between bone metastasis and all other locations combined achieving AUC of 74.6% for XGBoost, and it differed significantly from the Zhong^16^ study, which focused on bone metastasis as a primary outcome and utilized metastasis to other sites as a feature. The distinct advantage of our study lies in its comprehensive external validation using real patient data from Baheya Hospital. This validation step underscores the practical relevance and reliability of our models, ensuring their applicability in real-world clinical settings. One significant limitation in our study is the geographic and demographic focus of our training data, which was solely based on populations in the United States. The absence of datasets representing the Egyptian population prevented us from training our models on more region-specific data, which might limit the applicability and effectiveness of our predictive models in the Egyptian healthcare context. This study represents significant progress in precision oncology, offering hope and better outcomes for breast cancer patients globally by applying machine learning approaches on clinical data to develop and validate unique predictions for breast cancer recurrence types and metastasis, leveraging survival analysis. Our findings underscore the significant potential of these models to transform clinical practice, offering remarkable accuracy and robustness, particularly when validated with real-world data from the Baheya Foundation. This highlights their practical relevance and potential for immediate clinical application. Our study stands out as it encompasses all aspects of breast cancer recurrence prediction through three innovative approaches: assessing the risk of recurrence, distinguishing between local and distant recurrences, and predicting the likelihood of metastasis to specific sites, including bone, brain, liver, and lung. Our major findings indicate the key prognostic factors that are critical in predicting breast cancer recurrence. Expanding the dataset to include more diverse populations will enhance the generalizability and accuracy of our models. Our comprehensive approach to predicting recurrence sets a new benchmark, highlighting the value of data-driven strategies in improving cancer treatment. Looking ahead, future work will aim to incorporate genetic data to further refine these predictive models and address the current limitations.

**Fig. 6:**
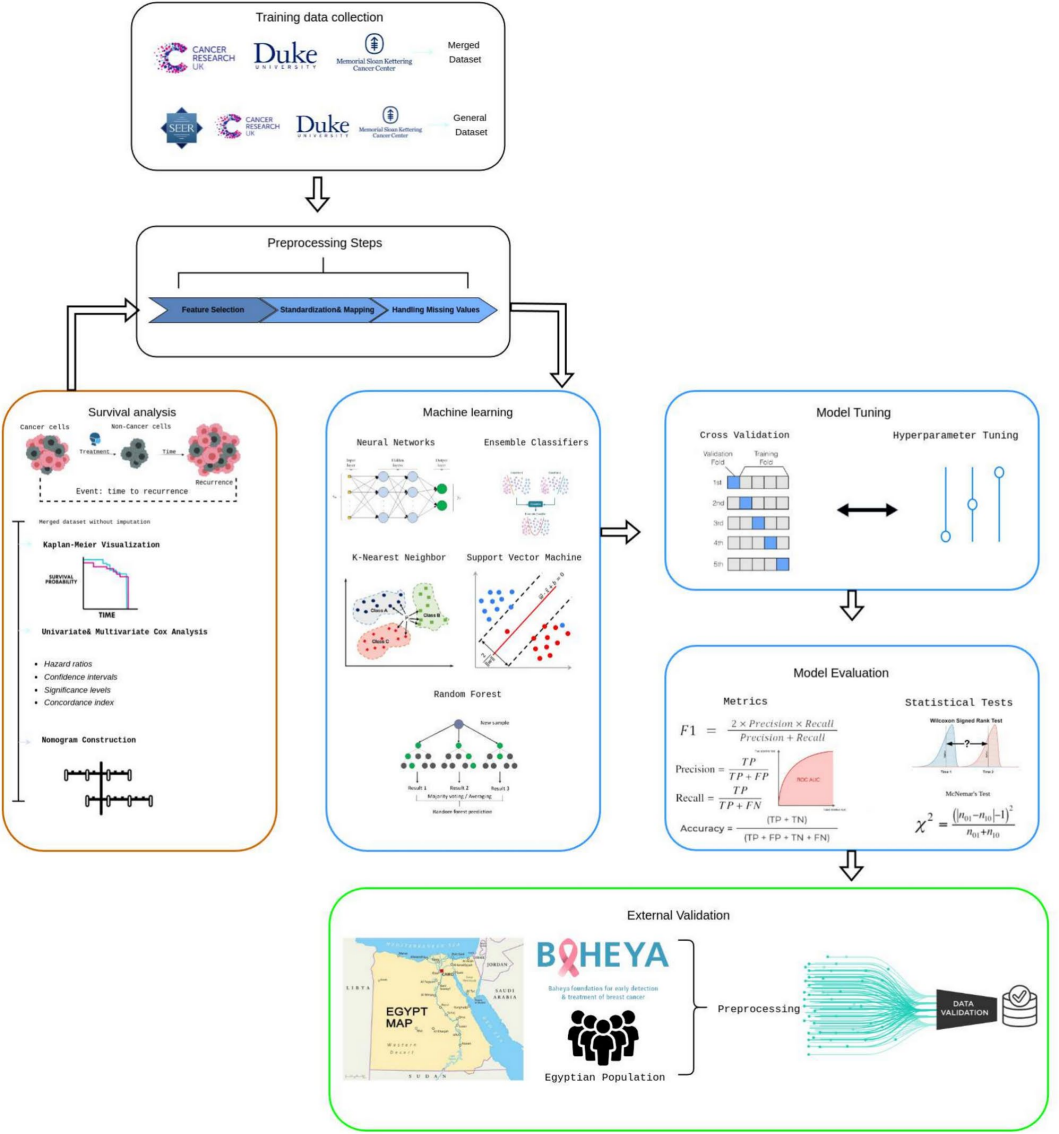
Workflow diagram of the comprehensive methodology from data collection to validation. This diagram outlines the process starting with the aggregation of training data from multiple sources, followed by detailed preprocessing steps. The methodology incorporates survival analysis and diverse machine learning techniques for outcome prediction, with subsequent hyperparameter tuning and model evaluation. The final phase involves external validation with population data to ensure model robustness.

## Methods

The research methodology is designed to enhance the prediction of breast cancer recurrence. Systematic data collection is followed by recurrence-free survival analysis to identify key predictors. The process includes rigorous feature engineering and data preprocessing. Models for binary classification of recurrence, type prediction, and identification of distant recurrence sites are developed and tested, with each model rigorously evaluated to ensure clinical reliability and accuracy. This approach aims to significantly improve management strategies for breast cancer recurrence. The detailed methodology is shown in Fig. 6 below.

### Data collection

**Fig. 7:**
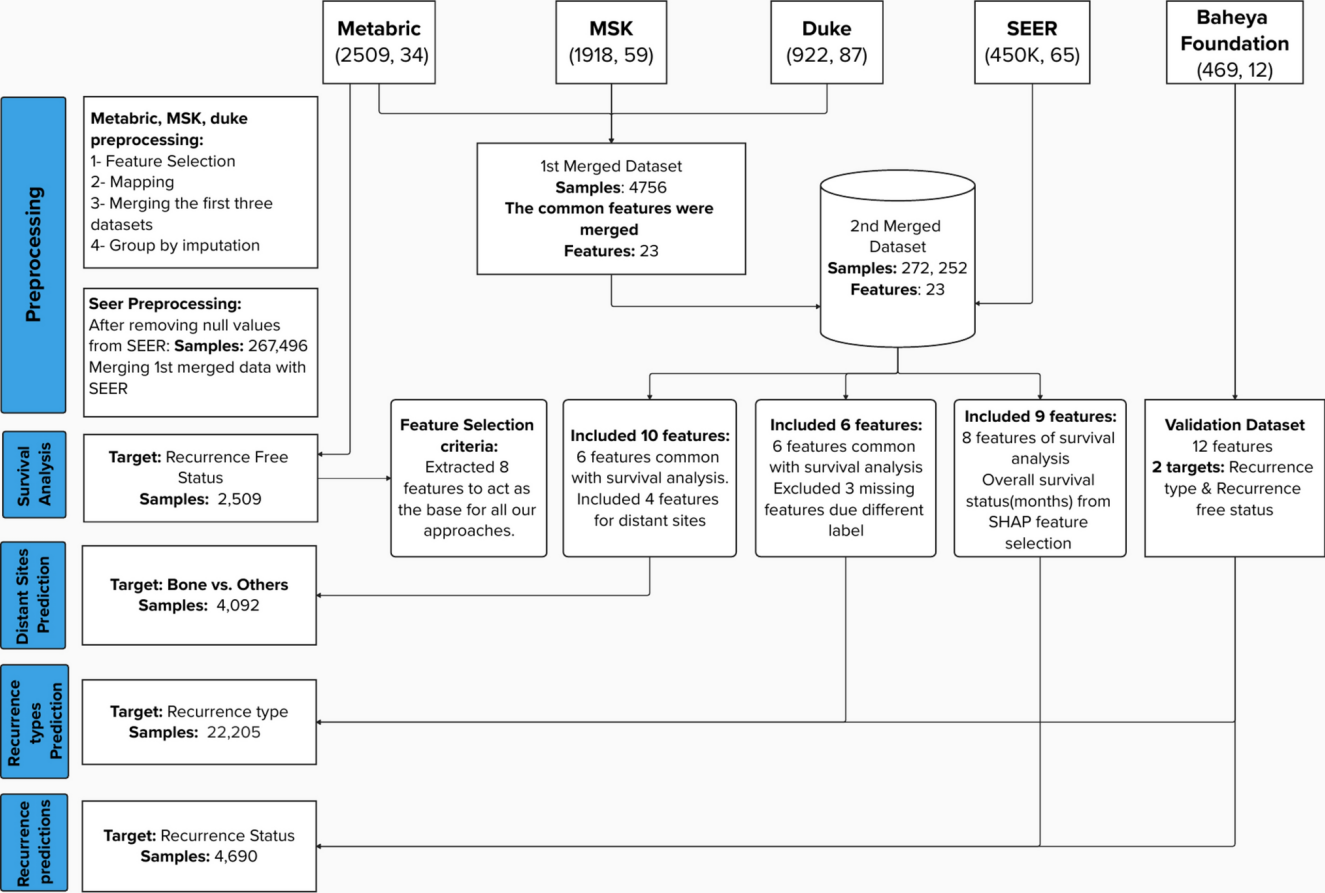
Overview of data preprocessing and integration pipeline: datasets from Metabric, MSK, Duke, SEER, and the Baheya Foundation are preprocessed, merged, and feature-selected to form comprehensive datasets for recurrence type, recurrence status, and distant site prediction approaches.

Data collection began by merging three datasets: METABRIC from cBioPortal, which includes records from 2,509 breast cancer patients^25^; the MSK dataset from cBioPortal, with 1,918 patients^26^; and the Duke University dataset from The Cancer Imaging Archive, containing approximately 922 patients^27^. The first merge combined these datasets into a single dataset with 4,756 rows and 23 columns. This merge was performed to enable group-by imputation, allowing missing values in one dataset to be filled using corresponding features from the other datasets. To enhance the dataset further, the SEER dataset was incorporated, covering breast cancer patients diagnosed between 2010 and 2016 and including data from 450,000 patients. This dataset was obtained from SEER cancer registries across the United States^16^. After filtering, the four merged datasets resulted in a comprehensive dataset with 272,252 rows and 23 columns, which was used to create the general pipeline. The exclusion criteria were based on aligning the common features between the four datasets to ensure compatibility with the validation dataset from the Baheya Foundation. Only overlapping clinical and pathological features available in all datasets were retained to maintain consistency. All details on the selection of features for each approach are provided in Fig. 7. For each specific approach, appropriate features from this general dataset were selected, as detailed in Table 3.

For external validation, anonymized patient data on real breast cancer cases was provided by the Baheya Foundation^5^. After filtering, 316 recurred patients (49 local and 267 distant) were identified, including four distant sites (bone, brain, liver, and lung). Additionally, 152 non-recurred patients were included. The Baheya Cancer Registry complies with the guidelines set forth by SEER. The validation data was used in two approaches: binary recurrence and local vs. distant recurrence classification This study was approved, and the need to obtain informed consent was waived by the Ethics Committee of Baheya Center (IRB202309250042) and conducted in accordance with the ethical principles of the Declaration of Helsinki, 1994. The study utilized de-identified retrospective data, ensuring that no participants could be directly or indirectly identified. As no images or information that could lead to the identification of individual participants were used, specific consent for publication was not required. Patient privacy and confidentiality were rigorously maintained throughout the research.

**Table 3:** Description of clinical and pathological features used in breast cancer prognosis and recurrence prediction.

Feature	Value	Binary rec	Local Vs. Distant	Distant sites
Menopausal status	Pre, Post	$\checkmark$		
Tumor size	T0–T4	$\checkmark$	$\checkmark$	$\checkmark$
Lymph node status	N0–N3	$\checkmark$	$\checkmark$	$\checkmark$
Tumor grade	I–III	$\checkmark$	$\checkmark$	$\checkmark$
Estrogen receptor (ER)	+, −	$\checkmark$	$\checkmark$	$\checkmark$
HER2	+, −	$\checkmark$	$\checkmark$	$\checkmark$
Overall survival (Month)	Numeric	$\checkmark$		
Rec free status (Month)	Numeric	$\checkmark$		
Age at diagnosis	Cat			$\checkmark$
Chemotherapy	Y, N			$\checkmark$
Radiotherapy	Y, N			$\checkmark$
Surgery	Y, N			$\checkmark$
Mol subtype	LumA, LumB, TNBC, HER2-E	$\checkmark$	$\checkmark$	$\checkmark$

### Recurrence-free survival analysis

The RFS included 2509 breast cancer patients, with 1403 experiencing recurrence. Missing values were excluded from the merged dataset to ensure data integrity. The primary event of interest was recurrence-free status, measured as the duration from diagnosis to relapse. Initially, Kaplan-Meier curves^28^ were used to visually depict survival probabilities over time for different groups. Subsequently, the significance was assessed using log-rank tests (p $< 0.05$) to compare survival curves across different covariates, identifying significant predictors of RFS. Following this, univariate analysis was conducted using Cox proportional hazards regression^29^ to evaluate the association between RFS and individual covariates.

Multivariate analysis was then performed to explore the combined effects of covariates on RFS. The Cox model estimated hazard ratios, confidence intervals, and significance levels, enabling the identification of influential factors. Model accuracy was assessed using the Concordance index. In addition to the regression analysis, a nomogram was constructed based on the significant covariates identified from the Cox model. This nomogram served as a practical tool for predicting 5-year RFS. Specialized R packages, including survival^30^, ggplot2^31^, and survminer^32^, were utilized for advanced survival analysis, visualization, and model construction aimed to identify key risk factors influencing RFS and providing practical tools for risk assessment in clinical practice.

### Feature engineering and data preprocessing

The datasets mentioned in section Data Collection were used for different approaches with various aims to predict the Recurrence Occurrence, Recurrence Types, and Metastasis Locations. Common columns were used to integrate the datasets, and rows lacking values in important fields were eliminated. Suitable procedures were employed to preserve data integrity while addressing missing data, with imputation carried out in accordance with group-specific features. As previously discussed in section Recurrence-Free Survival Analysis, the most crucial features were chosen based on the risk factors of the recurrence-free survival analysis. Feature selection for the three predictive approaches was strategically informed by survival analysis, ensuring the models leveraged the most relevant predictors. For the binary recurrence prediction approach, only features directly tied to recurrence-free survival status were selected, as this status was the primary target of analysis. The local vs. distant recurrence approach also relied on survival analysis but required omitting certain features unavailable in the specific sub-dataset labeled for this task, such as menopausal status and the overall survival (months). For metastatic site prediction, the base features from survival analysis were augmented with four additional predictors-Age, Surgery, Radiotherapy, and Chemotherapy-identified through a comprehensive review of relevant literature as recommended by zhong et al.^16^, tailored to enhance accuracy for this distinct predictive goal. Categorical variables were encoded using one-hot encoding, while numeric features were standardized using StandardScaler to ensure comparability across different scales. The target column changed according to each approach. For the binary recurrence prediction approach and the recurrence type approach, the sub-dataset was divided into 70% training and 30% testing sets, along with an external dataset for validation acquired from Baheya Foundation. While for the distant sites prediction approach, the merged dataset was divided into 80% training, 20% testing.

### Breast cancer recurrence binary classification

In this approach, a machine learning methodology was employed to predict the recurrence likelihood of breast cancer. The analysis was conducted using the first three merged datasets with the preprocessing mentioned in section preprocessing. The target unique values included: Not Recurred: 2579, Recurred: 2111. Five machine learning models were implemented and optimized using grid search to identify the best hyperparameters for each model, and the results are mentioned in Supplementary Table S1.

### Breast cancer recurrence types prediction

Using this method, the risk of breast cancer local recurrence or distant metastasis is predicted. The general dataset was processed using the preprocessing described in section preprocessing, with the features listed in Table 3. Due to a significant imbalance in the classes of the target (local: 170,745 and distant: 11,024), this issue was addressed through data balancing by applying a random down-sampling strategy to ensure a balanced representation of local and distant recurrence cases. The balanced dataset included 11,024 distant and 11,181 local instances. Multiple models were trained, optimizing each model’s hyperparameters using grid search with cross-validation. The best hyperparameters for each model are mentioned in Supplementary Table S1.

### Distant recurrence sites prediction

In this approach, a two-step methodology was implemented to determine the most effective strategy for predicting distant metastatic sites. As an initial step, a multi-class classification model was developed to predict the metastatic site of breast cancer that can be categorized into four distinct classes: bone, brain, liver, and lung. To address class imbalance, a random downsampling technique was employed, ensuring each category had 100 samples to match the least represented class, which is brain.

Additionally, Principal Component Analysis (PCA) was conducted using the R programming language to visualize the distribution of the four metastatic sites and evaluate whether they could be effectively separated in the feature space. The analysis utilized with R packages such as ggplot2^31^ and FactoMineR^33^, which facilitated data visualization.

For the second step focusing on binary classification models. The aim was to predict bone metastasis versus all other metastatic sites combined (lung, liver, and brain). The same dataset was used, but the target variable was modified to reflect the binary classification task, with the two classes labeled as Bone and Others. The samples were 2200 for bone and 1892 for other locations; the weighted sampling was applied to handle the imbalanced classes. In both steps, the dataset was split into training and testing sets with an 80:20 ratio, ensuring stratification to maintain the proportion of each class. Models used in this approach were LGBM, Randdom Forest, XGBoost and SVM. Furthermore, hyperparameter tuning was performed to optimize the models and achieve the best possible performance for the classification tasks.

### Model evaluation

A variety of classification metrics were employed, such as accuracy, precision, recall, F1-score, and AUC-ROC. The goal is to minimize false negatives and optimize the balance between sensitivity and specificity, since it is essential for medical predictions^34^. Additionally, to assess the significance of the observed differences in model performances, statistical testing was performed using methods such as the Wilcoxon test^35^, calculating the p-value models.

**Accuracy:** Measures the proportion of correct predictions out of the total predictions made by the model, indicating overall correctness.

$$\begin{aligned} Accuracy = \frac{TP + TN}{TP + TN + FP + FN} \end{aligned}$$

**Precision:** Represents the model’s ability to correctly identify positive instances from all instances predicted as positive, indicating the model’s precision in positive predictions.

$$\begin{aligned} Precision = \frac{TP}{TP + FP} \end{aligned}$$

**Recall (Sensitivity):** Reflects the proportion of actual positive instances that were correctly identified by the model, demonstrating the model’s sensitivity to detecting positives.

$$\begin{aligned} Recall = \frac{TP}{TP + FN} \end{aligned}$$

**F1-score:** Calculates the harmonic mean of precision and recall, offering a balanced assessment of a model’s performance in terms of both precision and recall.

$$\begin{aligned} F1-score = 2 \times \frac{Precision \times Recall}{Precision + Recall} \end{aligned}$$

**AUC-ROC:** Evaluates the model’s performance across various classification thresholds by plotting the true positive rate against the false positive rate, providing a single metric summarizing the model’s discriminatory ability.

$$\begin{aligned} AUC-ROC = \int _{0}^{1} \text {TPR}(fpr) \, d(fpr) \end{aligned}$$

## Supplementary Information

Supplementary Information.

## Data Availability

The datasets used and/or analysed during the current study available from the corresponding author on reasonable request.

## Code availability

The code to reproduce the pipeline from this work can be found at GitHub: (https://github.com/SarahElmaasarawii2131/BC).
