# Supplementary material for: Leveraging survival analysis and machine learning for accurate prediction of breast cancer recurrence and metastasis
Source: Sci Rep. 2025 Jan 29;15:3728. doi: 10.1038/s41598-025-87622-3 (PMC11779859; doi:10.1038/s41598-025-87622-3)
Supplement: Supplementary file 1 — Supplementary Information. [file 41598_2025_87622_MOESM1_ESM.docx]

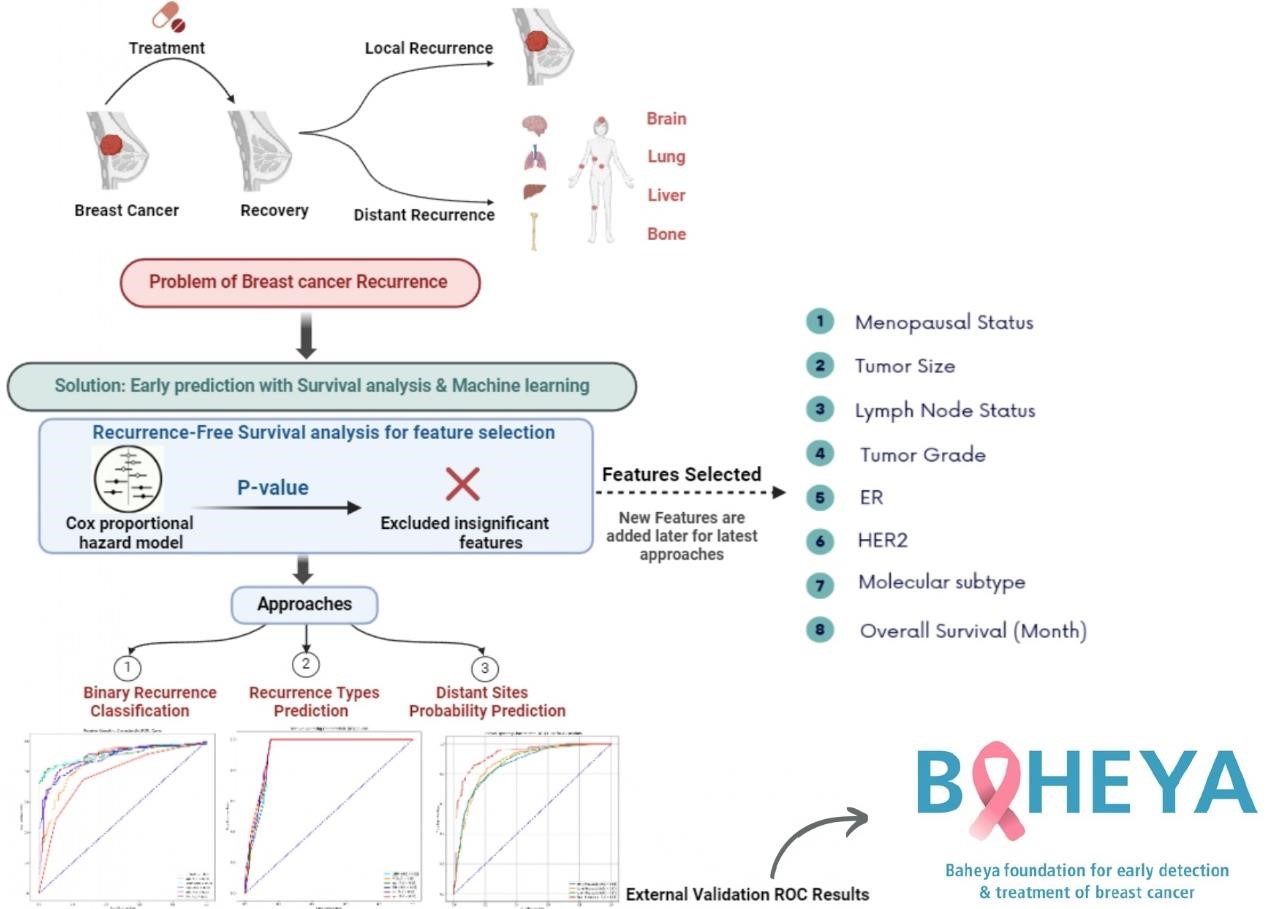


**Fig S1:** Abstract figure for predicting Breast Cancer Recurrence. Illustration of the methodological approach integrating clinical data and machine learning to predict breast cancer recurrence, featuring survival analysis for feature selection and model validation using ROC results, supported by the Baheya Foundation.

**Table S1**: Optimal Parameters for Machine Learning Models


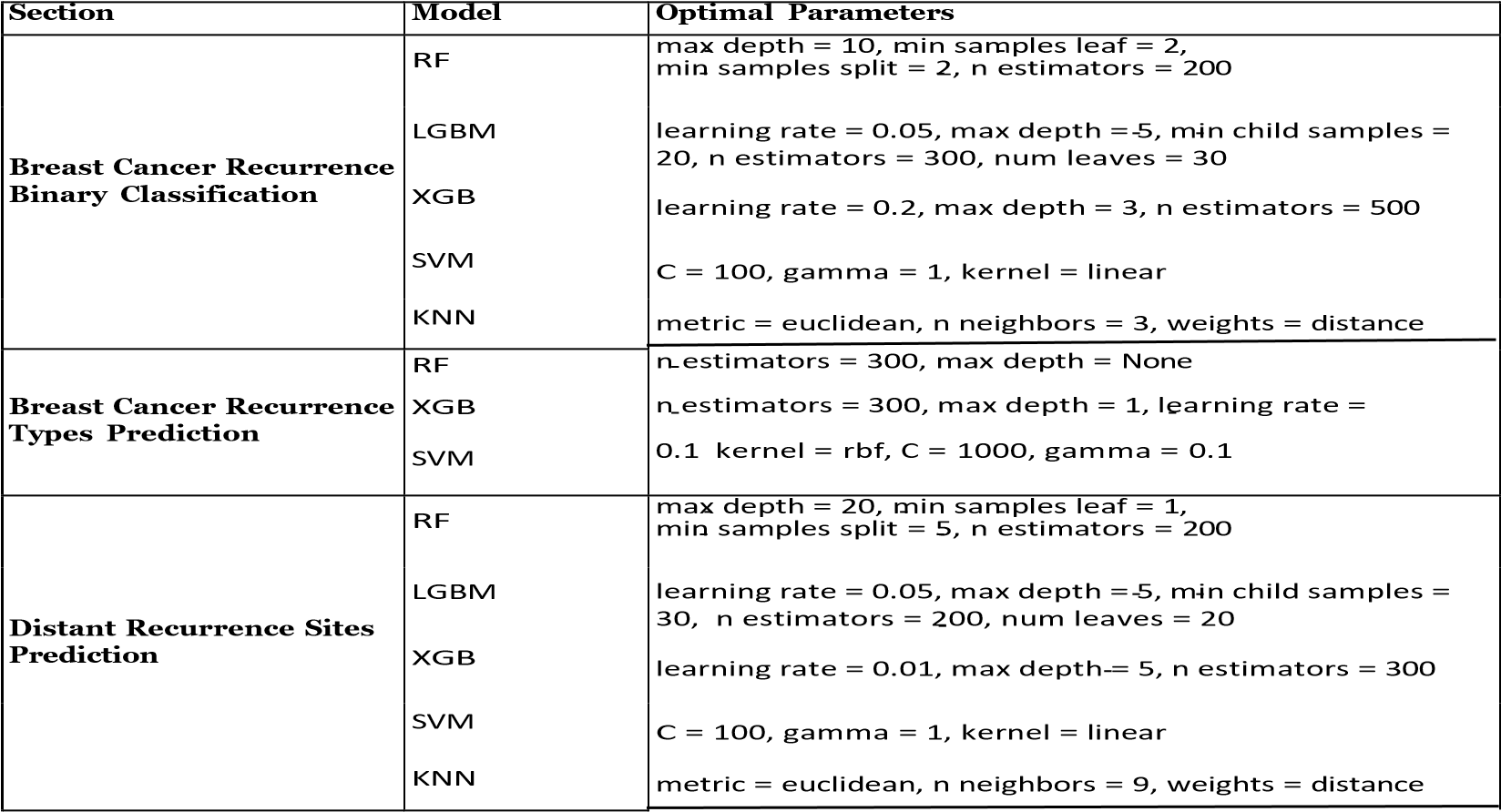


**Table S2**: Comparison of Our Findings with Previous Publications

| **Approach** | **Publication** | **Sample Size** | **Algorithm** | **Model**  **Evaluation** | **Validation Strategy** |
| --- | --- | --- | --- | --- | --- |
| **Recurrence**  **Binary**  **Classification** | Zuo and Yang  [10]    Our Publication | 342      4690 | AdaBoost      LGBM | AUC: 0.98      AUC: 0.92 | 3-fold cross- validation    Real Patients from  Baheya |
| **Recurrence**  **Types Prediction** | Chakkouch  [12]      Our Publication | 1,189        22,205 | ANN        RF | AUC: 0.97        AUC: 0.93 | 10-fold cross  validation      Real Patients from  Baheya |
| **Distant**  **Recurrence**  **Sites Prediction** | Zhong [14]      Our Publication | 3.492      13, 400 | XGBoost      LGBM | AUC: 0.80      AUC: 0.86 | 5-fold cross- validation    Real Patients from  Baheya |
